# Reasons for patients in high income countries accessing hospital care while receiving specialist community palliative care: A systematic review and meta-ethnography

**DOI:** 10.1177/02692163261418625

**Published:** 2026-02-25

**Authors:** Norah Elvidge, Melanie Rolfe, Karen Smith, Hannah E. Carter, Jane L. Phillips

**Affiliations:** 1 School of Nursing, Faculty of Health, Queensland University of Technology, Kelvin Grove, QLD, Australia; 2 Silverchain, Melbourne, VIC, Australia; 3 School of Population Health, Curtin University, Perth, WA, Australia; 4 Department of Epidemiology and Preventive Medicine, Monash University, Melbourne, VIC, Australia; 5 Australian Centre for Health Services Innovation and Centre for Healthcare Transformation, Brisbane, QLD, Australia; 6 School of Public Health and Social Work, Queensland University of Technology, Brisbane, QLD, Australia; 7 IMPACCT (Improving Palliative, Aged and Chronic Care through Clinical Research and Translation), University of Technology Sydney, Sydney, NSW, Australia

**Keywords:** palliative care, meta-ethnography, community health services, caregivers, systematic review, qualitative research

## Abstract

**Background:**

People receiving specialist community palliative care services often experience unplanned hospitalisations. A better understanding of the determinants leading to hospital use will support the development of more responsive palliative care policies and practices.

**Aim:**

To identify the contextual and individual determinants that lead to the unplanned hospital use of people receiving specialist community palliative care services.

**Design:**

A systematic review and meta-ethnographic synthesis prospectively registered with PROSPERO (CRD42022287904) and reported according to the PRISMA Statement and eMERGe guidelines. Andersen’s Behavioural Model of Health Service Use was used to frame the synthesis.

**Data sources:**

CINAHL, PubMed via the CareSearch palliative care filter, Scopus and Embase were searched in February 2024. High-income country’s studies published in English and reporting empirical qualitative data were eligible for inclusion.

**Results:**

The 12 included studies provided carers (*n* = 148) and healthcare professionals (*n* = 150) but not patients (*n* = 0) perspectives of factors contributing to unplanned hospital use. Five constructs were the key reasons for specialist community palliative care patients’ unplanned hospital use, including service limitations and barriers, difficulty accepting death, transitions as catalysts for misunderstanding, carer burden and hospital preference. The line of argument synthesis revealed convergence across studies and showed the decision to seek hospital care is multifaceted, shaped by individual and contextual factors, culminating in unplanned hospital use.

**Conclusions:**

This meta-ethnography identified insights for developing effective person-centred initiatives aimed at reducing unplanned hospital use and opportunities for specialist community palliative care services to support patient and carer needs. Capturing patient perspectives is essential for future research.

**What is already known about the topic?**

- Many people with palliative care needs would prefer to be cared for and/or die at home.
- Many people will be cared for at home by specialist community palliative care services.
- Despite this, hospitalisation rates remain high for people receiving specialist community palliative care services.
- Carers face physical, emotional and financial burden caring for someone with palliative care needs at home.

**What this paper adds?**

- Provides a deeper understanding of the contextual and individual determinants of why people receiving specialist community palliative care access unplanned hospital care.
- Provides insight into specialist palliative care carer behaviour at the end of life.
- Highlights the importance of the carer’s role in decisions surrounding acute care access for people at the end of life.

**Implications for practice, theory or policy**

- Further research is needed to capture the perspective of people receiving specialist community palliative care regarding the reasons for unplanned hospital use.
- The number of unplanned hospital presentations for people receiving community palliative care may be reduced through the provision of better formal support for their carers.
- Examining factors contributing to unplanned hospital use supports specialist community palliative care services to refine models of care and optimise care delivery.

## Introduction

### Background

Globally, there is a need to understand the impact of specialist community palliative care on patients’ unplanned acute emergency and hospital (‘hospital’) use.^1^ Although primary palliative care is a fundamental responsibility of all healthcare providers and services, specialist palliative care supports individuals with advanced progressive illness when their physical, psychosocial or spiritual needs exceed the expertise of their usual care team.^2–4^ Provided by a multidisciplinary team with core palliative care competencies and skills to help meet the patient’s goals,^5,6^ it is available in inpatient and community settings (e.g. private homes and nursing homes).^7,8^ Between 20% and 80% of people would prefer to die at home, with most studies suggesting this is the preference for more than 50%.^9–14^

Despite this preference, unplanned hospital use remains prevalent in the final year of life among the general population, with 78%–91% of individuals experiencing at least one hospital admission during this period.^15–18^ One of the aims of specialist community palliative care is to reduce the frequency of hospital use at the end of life, and while numerous observational studies have reported a reduction compared to the general palliative population,^3,19–30^ this impact has not been demonstrated in adequately powered randomised controlled trials.^31–33^ A 2022 systematic review concluded that specialist community palliative care significantly reduced hospital admissions in only half of the included studies.^28^ Even when a reduction was significant, 15%–68% of those receiving specialist community palliative care still accessed the emergency department in the last months or weeks of life.^28^

The existing literature regarding the unplanned hospitalisation of specialist community palliative care patients primarily focuses on the rates and/or length of admission and place of death.^28,34^ There is a lack of synthesis regarding the reasons for this cohort accessing hospital services.^29,35–38^ Few of these studies critically examined the motivations of community palliative care patients, their informal carers (‘carers’) or specialist palliative care teams for accessing unplanned acute care.

While the data on acute care service utilisation is valuable, a more comprehensive understanding of healthcare access encompasses the *‘. . .actual use of personal health services and everything that facilitates or impedes their use*’ (p. 33–34).^39^ Accordingly, Andersen et al. affirm that both contextual and individual determinants frame the facilitation and impediments of health service access; *contextual* determinants shape healthcare use at a systemic level, while *individual* determinants, including beliefs, knowledge, and socio-economic factors, influence personal health perceptions and access to care.^39^ Understanding and synthesising these determinants is critical to strengthening policy and service design that may support people with palliative care needs living at home to spend more days in their preferred place of care.

This study aims to identify the contextual and individual determinants that lead to unplanned hospital use of people receiving specialist community palliative care services.

### Method

A systematic review and meta-ethnography (‘meta-ethnography’) was conducted. Meta-ethnography is an inductive qualitative research method that uses iterative data translation and synthesis to develop conceptual and theoretical insights.^40,41^ Reinterpreting the primary data through a meta-ethnographic approach allows for the development of new theories, concepts and models that can be used to generate evidence for healthcare practice and policy.^40–42^ Meta-ethnography is commonly used in healthcare research, including recent research that has developed conceptual models and a deeper understanding of palliative care practices.^43–46^ The meta-ethnographic process developed by Sattar et al.,^41^ grounded in Noblit and Hare’s original 1988 seven-step methodology,^40^ was followed (Refer to Textbox 1). To ensure standards for both systematic review and meta-ethnographic synthesis were met, this meta-ethnography was registered with PROSPERO (CRD42024495016) and reported according to the PRISMA Statement,^47^ in addition to the eMERGe guidelines for meta-ethnographic synthesis.^42^

**Textbox 1.** Phases of Meta-Ethnography, initially developed by Noblit and Hare^40^ and operationalised by Sattar et al.^41^.

Phase 1: Getting started	Identifying if a meta-ethnographic approach is appropriate for the area of interest.
Phase 2: Deciding what is relevant to the initial interest	Defining study focus, identifying inclusion criteria and selecting studies and quality assessment.
Phase 3: Reading the studies	Re-reading studies and extracting first and second-order constructs.
Phase 4: Determining how the studies are related	An iterative process of grouping common concepts and themes.
Phase 5: Translating the studies into one another	Comparing themes and concepts inductively from one study to another.
Phase 6: Synthesising the translations	Developing third-order constructs via reciprocal or refutational synthesis and line of argument synthesis.
Phase 7: Expressing the synthesis	Following the eMERGE reporting guidelines^42^ for methodological robustness in reporting the synthesis.

### Search strategy

A list of base search terms was developed, consisting of terms related to home care, palliative care and hospital admissions (see Supplemental File 1). The search terms were then adapted individually for each database, and searches were systematically run on Embase, CINAHL, PubMed (using the CareSearch palliative care filter)^48^ and Scopus on February 8th, 2024. Search alerts were created and notified the primary author monthly with updated search results up to the date of submission.

### Inclusion criteria

This meta-ethnography considered studies published in English since 2014 in a peer-reviewed journal, originating from a high-income economy (as categorised by The World Bank)^49^ to ensure comparable healthcare systems. Qualitative studies of people of any age (e.g. neonates, paediatrics, adolescents, young adults, working adults and older people) with a life-limiting condition who are receiving specialist community palliative care services were considered eligible for inclusion. Studies had to include a proportion of patients where the reason (i.e. symptom, event, experience or belief) directly precipitating acute care service was recorded.

### Quality appraisal

The JBI suite of critical appraisal tools was used to assess the quality of the included studies.^50,51^ Two reviewers independently assessed each included study and discussed the merits and risk of bias inherent in each study according to the checklist questions. The JBI critical appraisal tools are not designed to facilitate a cut-off value for inclusion or exclusion; rather the intention is for authors to consider and determine inclusion themselves.^51,52^

### Study selection

Studies returned from the search were imported into the bibliographic management software *Covidence*™ which automatically removed duplicates. The research team (NE, MR, JLP, KS) reviewed the title and abstracts to ensure the article met inclusion criteria. Two reviewers (NE and MR) completed the full-text review individually, with any disagreements resolved via discussion. A third reviewer (JLP) was consulted for any remaining conflicts.

### Data extraction and translation

A proforma template was developed to extract study characteristics and data relevant to the study aim. The meta-ethnographic process commenced by sorting data into first-order constructs (participant direct quotes) and second-order constructs (individual study author interpretations).^40,42^ Two reviewers (NE and MR) completed extraction separately and resolved conflicts via discussion. Reciprocal translation was applied to the included studies, where the concepts and findings of one study are applied to a second, and then to each subsequent study; an iterative process that forms a common language across studies.^40,41^ Original studies were subsequently re-read to ensure participant quotes and author interpretations had not been taken out of their original context during translation.^42^

### Synthesis

The second-order constructs and translation are compared iteratively and re-interpreted by the study authors into higher-level themes, referred to as third-order constructs.^40,41^ These themes reflect the meta-ethnography authors’ interpretation of the original data, offering a more nuanced understanding of the reasons for unplanned hospital use than the concepts identified in individual studies alone.^40,41^ The synthesis process integrated first- and second-order constructs, and although some overlapped thematically, the final alignment of third-order constructs was reached through discussion until consensus was achieved.

The second stage of synthesis was what Noblit and Hare refer to as a ‘line of argument synthesis’;^40^ constructing a broader interpretive framework of reasons for hospital use by examining relationships between third-order constructs and uncovering new insights beyond the findings from the individual studies.^41,42^ Rather than summarising the third-order constructs, this process integrates them to illustrate how these themes interact within the broader system. While this is traditionally a purely inductive process, recent work by Noblit has suggested the overall intention of the synthesis is ‘. . .simply an interpretation of interpretations. . .’ (p. 6).^53^ Therefore, a deductive element was introduced by interpreting the third-order constructs through the lens of the Behavioural Model of Health Service Use by Andersen et al., (‘Andersen’s Model’),^39^ illustrating patterns of hospital use among specialist community palliative care patients. The authors’ expertise in palliative care, nursing and psychology was well-suited to interpret this model, generating explanations of hospital use in this population and presenting them in a manner accessible to real-world stakeholders involved in community palliative care service design. This synthesis was completed through discussion, in which the authors reviewed translation and synthesis findings to ensure the preservation of conceptual richness.

#### Andersen’s behavioural model of health service use

Since its inception in 1968, Andersen’s Model has evolved into a person-centred framework for assessing healthcare access and utilisation.^39^ The model comprises contextual determinants that influence a person’s environment and individual determinants that shape health behaviours and service utilisation, ultimately affecting health outcomes.^39^ Andersen et al. argue that identifying and addressing both contextual and individual determinants of healthcare access is essential for driving systemic improvements.^39^

## Results

### Study selection

**Figure 1.**
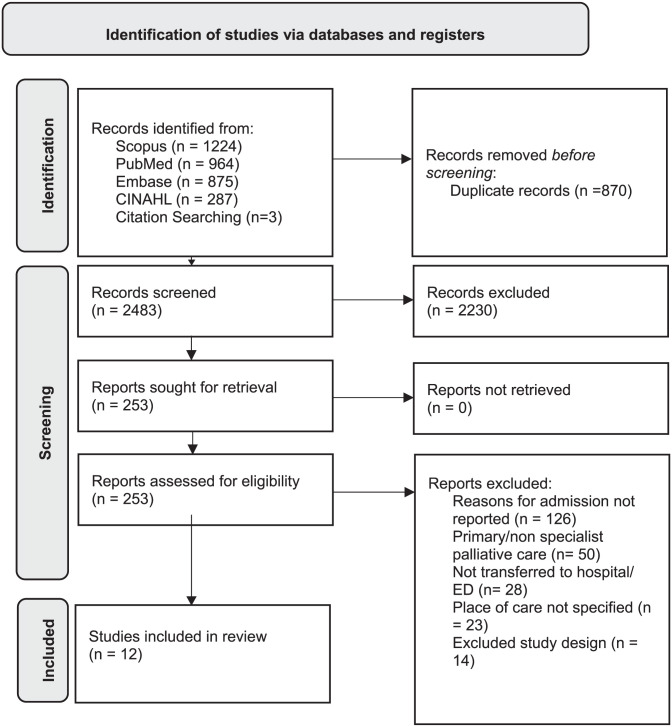
PRISMA flowchart.

Of the 3353imported citations, 870 duplicates were removed, and after title and abstract review, 253 articles were extracted for full-text review, with 12 assessed as meeting the inclusion criteria. A complete breakdown of the study selection is illustrated in the PRISMA flowchart (Figure 1). All included studies were determined to be of acceptable quality.

### Study characteristics

The 12 included studies originated from six countries, with most from the United States (US) (*n* = 5) and Australia (*n* = 3) (Table 1). The studies primarily represented patients who lived in their own, or a carer’s home, though three studies^54–56^ defined a patient’s home as including a nursing home setting.

**Table 1.** Characteristics of included studies (n = 12).

Author/Date/Country	Participant type	Demographics	Primary diagnosis	Description of specialist service	Data source
Yoon et al. (2024), Singapore	Carers of adult patients (*N* = 30).	x̄ age 46.1(SD 9.7), 73% female.	Cancer (100%).	Specialist community palliative care provided in the patient/carer home.	Interviews.
Halevi Hochwald et al. (2022), Israel	Healthcare professionals (*N* = 13).	Median age 47, 85% female.	Dementia/Frailty (100%).	Specialist community palliative care provided in the patient/carer home	Interviews.
DeAngelis and Lowry (2021), United States	Adult patients (*N* = 75).	x̄ age 69.2(SD 15), 61% female.	Cancer (46%, *n* = 35), dementia (15%, *n* = 11), heart failure (11%, *n* = 8), lung disease (11%, *n* = 8), other (17%, *n* = 13).	Specialist community palliative care provided in the patient/carer home or nursing home.	Chart review - progress notes.
Papadatou et al. (2021), Greece	Parent/carers of 22 paediatric patients (*N* = 36).	56% female.	Malignant (73%, *n* = 16), non-malignant (27%, *n* = 6).	Specialist community palliative care service provided in the patient/carer home.	Interviews.
Ward et al. (2021), United Kingdom	Carers (*n* = 38) and healthcare professionals (*n* = 9).	Not reported.	Not reported.	Specialist community palliative care provided in the patient/carer home.	Interviews.
Russell et al. (2019), United States	Healthcare professionals (*N* = 19).	x̄ age 48.8(SD 12.3), 79% female.	Cardiovascular disease (100%).	Specialist community palliative care provided in the patient/carer home	Interviews.
Phongtankuel et al. (2017), United States	Carers of adult patients (*N* = 38).	66% female.	Cancer (60%, *n* = 23), noncancer (40%, *n* = 15).	Specialist community palliative care provided in the patient/carer home.	Interviews.
Phongtankuel et al. (2016), United States	Healthcare professionals (*N* = 73)	Not reported.	Not reported.	Specialist community palliative care provided in the patient/carer home.	Focus groups.
Batchelor (2015), United States	Nurses (*N* = 21).	47% < 50 years and 53% > 50 years.	Cancer, dementia/frailty, respiratory disease, heart/vascular disease, adult failure to thrive (*n* = not reported).	Specialist community palliative care provided in the patient/carer home or nursing home.	Survey with free text responses.
Champion (2015), Australia	Adult patients (*N* = 14).	Not reported.	Not reported.	Specialist community palliative care service provided in the patient/carer home.	Case note review.
Lane and Philip (2015), Australia	Healthcare professionals (*N* = 15).	93% female.	Not reported.	Specialist community palliative care service provided in the nursing home.	Interviews and focus groups.
Hatcher et al. (2014), Australia	Carers (*N* = 6).	Age range 46–72, 50% female.	Cancer (100%).	Specialist community palliative care service provided in the patient/carer home.	Interviews.

The included studies presented data from healthcare professionals working in specialist community palliative care (*n* = 150)^54,55,57–60^ and carers (*n* = 148).^58,61–64^ While adult patient study populations were referred to in two primary studies (*n* = 89),^56,65^ the qualitative findings were taken from progress/case notes, and the content was interpreted by the healthcare professional writing the entries. As a result, the patient perspective is not directly represented in this meta-ethnography. The five studies that interviewed carers^58,61–64^ did not include patients in their recruitment strategy, meaning that the absence of patient perspectives was not due to recruitment challenges, but rather an exclusion from the methodological approach. Of the eight (75%) studies reporting diagnosis, cancer was the most common primary diagnosis for people with palliative care needs.^54,58,60,65^ Two studies^56,64^ did not report any first-order constructs (direct participant quotes), and the reasons for hospital use, were solely extracted as second-order constructs.

### Translation and synthesis result

**Figure 2.**
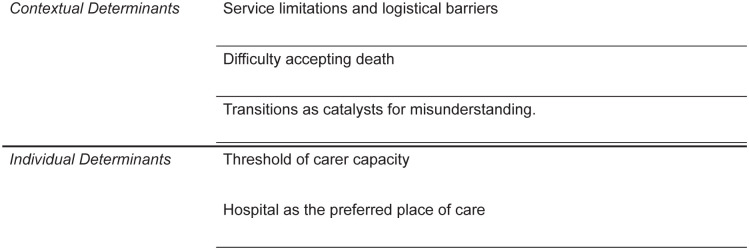
Third-order constructs generated through the meta-ethnographic process.

The synthesis generated five third-order constructs distributed across two overarching conceptual domains, as depicted in Figure 2. Guided by Andersen’s Model, the first domain relates to contextual determinants, and the second to individual determinants. Table 2 provides an overview of the first, second and third-order constructs generated in this meta-ethnography. While the first and second-order constructs differentiated the perspectives of carers and healthcare professionals, the third-order constructs ultimately combine all perspectives due to the reciprocal nature of the synthesis.

**Table 2.** Summary of the meta-ethnographic translation and synthesis process.

Third-order constructs (meta-ethnography authors’ interpretation)	First-order constructs (participant quote)	Second-order constructs (primary study author interpretation)
*Contextual determinants*		
Service Limitations and Logistical Barriers	Carer Perspective: ‘Unfortunately, there were multiple instances when his pain was poorly managed [by the specialist palliative care service]. We called the (homecare) nurse and wanted to increase the morphine dosage, but the [specialist palliative care service] doctors were reluctant. We wanted to know why but did not get explanations. In the end, we had no choice but to call ambulances’. (Carer, p. 4)^61^ ‘I wished [hospice service] could’ve provided us with more options to ease the burden of caregiving, like private nurses, home medical services, or even respite care’. (Carer, p. 5)^61^ ‘I think one of the factors that precipitated the frequent hospital admissions was the absence of home support. [specialist palliative care service] support did not extend to address this aspect. Given my mom’s advanced age in her 80s, it posed a big challenge to care for my father. When he was unattended, he experienced several falls, so he was readmitted to the hospital . . . sometimes, I thought hospital admissions would be better because there are professionals who provide the care’. (Carer, p. 4)^61^	Carer Perspective: • Need for equipment in the home.^65^ • Difficulty transporting patient to appointments.^56^ • Lack of communication with patient/carers from specialist community palliative care team.^61^ • Carer seeking fastest response time.^63^ • Specialist community palliative care clinicians unable to meet patient needs due to: • Staff availability.^56,61^ • Scope of service.^61,63^
	Healthcare Professional Perspective: ‘The other part of that is we can’t get there fast enough for them, [families] panic, they want somebody, and something done right now. And so they’ll call us and if we don’t respond fast enough they’ll call 911’. (HCP, p. 54)^60^ [Patients with limited financial resources are often] ‘struggling to put something together’.(HCP, p. 554)^59^ ‘Most people that call [911] don’t have any support at home. They live alone. Not being able to breathe and live alone, have no one to call right?’ (HCP, p. 555)^59^	Healthcare Professional Perspective: • Limited/unavailable out of hours support.^65^ • Delay in service provision.^60,65^ • Appropriate hospital admissions due to limited scope of specialist community service.^54^ • Socioeconomic factors influence outcomes.^56,59^ • Social support correlated with patients remaining at home.^59^
Difficulty accepting death	Carer Perspective: ‘What I experienced at home was beautiful. . .but at the very last moment, I do not know what I felt, but I had to do something, and called for an ambulance’. (Carer, p. 226)^62^	Carer Perspective: • ‘Good parent’ is: saviour, guardian, advocate of their child, and protector of siblings.^62^
	Healthcare Professional Perspective: One concept included the ‘hero’, which was invoked to describe patients who ‘don’t want to die’ and associated aggressive measures, including hospitalisation, with ‘reassurance’ that they were taking every possible action to prolong life. (Primary author quoting HCPs, p. 554-555)^59^ ‘Some nursing homes. . .have a very good palliative approach. Others are still in that mindset of trying to keep people alive’. (Nurse, p. 78)^54^ ‘Patient/carers believe the hospital is a place that provides them with ‘every opportunity to live’. (HCP, p. 555)^59^	Healthcare Professional Perspective: • Prolonging life is heroic.^59^ • Carer not wanting to hasten death with medication.^59,60^ • Stigma of opioids is preventing effective symptom control.^60^ • Difficulty accepting their prognosis.^62^
Transitions as catalysts for misunderstanding	Carer Perspective: None reported.	Carer Perspective: • None reported.
	Healthcare Professional Perspective: ‘. . . caregiver brought a patient to the hospital for altered mental status because the caregiver thought hospice focused solely on wound care. . .these findings show the need for further studies that investigate these issues contributing to readmission’. (Derived from patient chart notes, p. 748)^56^ ‘Physicians. . .will [not] emphasize that. . .there’s really no more treatment: you can come back for A, B, and C [but] not for treatment. And I don’t think that’s always reinforced’. (Nurse, p. 53)^60^ ‘I found that I have a lot of patients from a [medical center who] have not been told that they are dying [. . .] I think if there was better patient education on that end, it would eliminate a lot of [hospitalization]’. (HCP, p. 53)^60^ [Patients and families who] ‘come into hospice not having any idea what it’s about’, ‘don’t understand the whole prognosis’, are ‘expecting a miracle’. (HCPs, p. 554)^59^ [patients believe] ‘if they call 911 and they go to the hospital. . .they feel that if they hear it from the hospital or doctor there, that’s different’. (Nurse, p. 554)^59^ ‘Unless you have got the [advance care planning] documentation there, you actually can’t do anything else but send them back [to hospital]’. (CPCS Nurse, p. 79)^54^	Healthcare Professional Perspective: • Providing palliative care in nursing homes is challenging due to acute medical model of care.^54^ • Patients/carers have a limited or poor understanding of palliative care philosophy.^56,59,60,62^ • Patients and/or carer are unaware of terminal prognosis^59,60^ • Patient/carers may have differing expectations than what palliative care provide.^60^ • Miscommunication in completing ACP documentation in nursing home.^54^
*Individual determinants*		
Threshold of carer capacity	Carer Perspective: ‘He seemed to suffer a lot because he couldn’t breathe properly. Even though the nurses had shown us how to use a nebulizer, we were overwhelmed with panic in that moment, so we made the decision to send him to the hospital’. (Carer, p. 4)^61^ ‘The moment I saw her suffering, her whole body was shivering, I was afraid as I am not medically trained. The [homecare] nurse informed us to call them anytime in the event of emergency, but when you see your loved one is suffering, you are compelled to take immediate action. You have to arrange for hospital transfer so she could receive prompt medical attention’. (Carer, p.4)^61^ ‘. . .he is a dead weight’, and ‘I can’t move him’. (Patient chart notes, p. 10)^65^ ‘My sister told me she turned blue. [She] panicked. . .[she] called 911 right away. . .I think it’s harder when your loved one dies at home. You’re right there, you’re with the person everyday, just to watch them die it’s not a good feeling’. (Carer, p. 262)^63^ ‘When I came [home] and saw [she was having a seizure], I said we have to call 911. . .it was horrific. . .’ (Carer, p. 262)^63^ ‘She fell, it was at night. . .She broke her hip’ (Carer, p. 262)^63^ ‘I’d had to phone 999 sometime before, because then he had to go into hospital with pneumonia’. (Carer, p.32)^58^ ‘. . .with the cancer, when you get to 38 degrees [temperature], then it’s a trigger point - so I’ve had an ambulance out’. (Carer, p. 32)^58^ ‘Carer reports client is ‘anxious and at times this exacerbates her breathlessness’ (Patient chart note, p. 10)^65^ ‘She fell out of bed and broke several vertebrae. She also needed stitches. I called the home hospice hotline first and when the nurse arrived, she advised me to call 911’. (Carer, p. 263)^63^ ‘My mother was experiencing intense pain. The prescribed medications didn’t provide much relief. Even when the [hospice service] came, there wasn’t much improvement. She desperately sought relief from the pain and wanted to go to hospital. It was at this point that I made a decision to transfer her to the hospital as I did not want her to feel abandoned or [that] we had given up on her’. (Carer, p. 3)^61^	Carer Perspective: • Carer distress, fear and anxiety.^56,64^ • Patient distress/anxiety.^64,65^ • Carer uncomfortable with patient dying in their care.^63^ • Carer uncertain of medication indication/administration.^65^ • Carer not prepared for patient to die.^63^ • Carers lacking informal support for themselves^61^ • Acute symptoms perceived as a ‘crisis’ by carers.^59,60^ • Manual handling difficulty.^65^ • Physical symptoms lead to acute service use, particularly dyspnoea, pain and change in cognitive status.^56,61,63–65^ • Acute events (i.e. fall, seizure).^61,63^
	Healthcare Professional Perspective: ‘Some families really are inept to take care of medical things. So they get in too deep, too quickly, they’re just not capable, and in fear, they send the patient to the hospital’ (Service Manager, p. 53)^60^ ‘So even though you teach [caregivers], when something happens and you tell them, well, you can give them morphine—‘I can’t do it, I need somebody else to do it’. And [caregivers] just 9-1-1 it, because that’s what they’ve done their whole life’. (HCP, p. 53)^60^ ‘They understand [the wish of the patient is to die at home], but at a moment of crisis when they see a loved one [dying], it’s like all bets are off. Sort of like, “You know, this is my loved one and I know they don’t [want to go to the ER] but I’m calling 911”. [Caregivers are] wrestling with the feeling of not doing the right thing for my loved one’. (Spiritual Care Counsellor, p. 54)^60^ ‘the symptoms could set on, it’s so sudden’ and ‘I could see that he had declined a lot from one time to the next’. (HCPs, p. 554)^59^ ‘Large necrotic area to both buttocks observed’. (Patient chart note, p. 10)^65^ ‘It’s a breathing problem. That’s why [caregivers] call 911 - they cannot get the patient comfortable breathing’. (Nurse, p. 53)^60^	Healthcare Professional Perspective: • Carer distress, fear and anxiety.^55,65^ • Carers turn to acute care when they realise they are not prepared to manage the patients’ needs.^60,65^ • Carers more likely to panic if trust with palliative care team is not established.^59^ • Physical symptoms are hard to witness for carers.^60,63^ • Complex physical and psychological needs at the end of life.^58,65^ • Disease specific physical characteristics.^59^ • Patient requires new medication (eg, antibiotics).^56^ • Patient medication dose too low to manage symptoms.^56^ • Other medication issues (non-compliance, dose too high, adverse reaction).^56,65^
Hospital as the preferred place of care	Carer Perspective ‘Because the hospital setting, her primary doctor is there and the people she knows are there and plus people are closer to her home where family members can either walk or travel by car to visit her’. (Carer, p. 262)^63^ Caregivers called 911 because [that is] ‘what we have always done’ or ‘wanted to see a physician’. (p. 445)^55^ ‘My father did not mind dying in the hospital, and he strongly preferred receiving medical care for his symptoms in the hospital . . . he experienced abdominal bloating, prompting me to call the [specialist palliative care service] nurse. After assessing, she suggested waiting until the following day for her visit. But my father insisted, so I had to call the ambulance’. (Carer, p. 3)^61^ ‘We had a routine appointment to see the cardiologist and he said that he had to keep her, and then sent her directly to the hospital’. (Carer, p. 263)^63^ ‘GP visiting later today but carer unsure if client could wait this long. Declined attending the GP surgery earlier if an appointment were available’. (Patient chart note, p. 10)^65^	Carer Perspective: • Shifting decisions at imminent end of life.^62^ • Preferencing known physicians.^63^ • Hospital as a default response to an unforeseen event.^61^ • Hospital as unavoidable^61^ • HCP recommends hospitalisation.^63^ • Patient preference or request for hospital care.^55,61,63^
	Healthcare Professional Perspective: ‘. . . still a strong relationship with the cancer centre where [patients] were being treated, and wanting to go back there or feeling more of a connection with them than with the hospice team’. (HCP, p. 53)^60^ ‘It takes time to build trust. When a patient first comes into our care, they are usually very unstable. If in the first week since we met there is fever in the middle of the night, the family will surely take him to the hospital. At the next visit I’ll tell the family to call us, even in the middle of the night the next time there is fever and that I’m a good reliable source they should trust. But they need to know me, my abilities . . . It takes time . . . If I get to know the patient in the last 24 h of life, he is unstable, in pain, breathing heavily . . . They don’t know me yet . . . sure, they will go to the hospital and he (the PWESD) will die there in the ER or in the intensive care unit’. (Nurse, p. 1673)^57^ [Patient/carers believe] the hospital is ‘where you go when you’re sick’ (HCP, p. 555)^59^ ‘. . .information regarding how to contact the hospice nurse when issues arise and not to call 911 or go to the ED for symptom management is given to patients and caregivers. However, even with instruction to call the hospice nurse for symptom management issues, patients still access the ED’. (Study author, p. 447)^55^	Healthcare Professional Perspective: • Existing relationships between patients and hospital clinicans.^57,60^ • Trust in clinicians takes time to develop.^57^ • Patient/carers accessing acute care despite being directed to call palliative care service first.^55,63^

ACP: advance care planning; ED: emergency department; GP: general practitioner; HCP: health care professional; PWESD: person with end stage disease.

#### Contextual determinants

The following third-order constructs relate to the external or systemic factors that influence access and decision making, including characteristics of the health system or service, organisational care transition practices and societal and/or cultural expectations and beliefs.

##### Service limitations and logistical barriers

Service limitations included equipment needs,^65^ staff availability,^56,61,65^ and limited scope or capacity to meet the person’s palliative care needs.^54,61,63^ Response time featured prominently, with carers and others reporting calling emergency services due to the faster response time compared to their local palliative care service:^60,63,65^

. . .we can’t get there fast enough for them, [families] panic, they want somebody, and something done right now. And so they’ll call us and if we don’t respond fast enough they’ll call 911 (HCP, p. 54)^60^

The practical barriers of remaining at home due to increasing manual handling demands or a lack of suitable in-home equipment are exemplified in excerpts relayed by carers from patient charts; ‘. . .he is a dead weight’ and ‘I can’t move him’ (Patient chart notes, p. 10).^65^ Carers, especially those who were older and felt unsupported, often sought hospital (re)admissions as a form of respite:

I think *one* of the factors that precipitated the frequent hospital admissions was the absence of home support. . .Given my mom’s advanced age in her 80s, it posed a big challenge to care for my father. When he was unattended, he experienced several falls, so he was readmitted to the hospital . . . sometimes, I thought hospital admissions would be better because there are professionals who provide the care. (Carer, p. 4)^61^

Limited access to external sources of formal support, beyond what is provided by specialist palliative care services, can present significant logistical barriers to remaining at home. A patient’s own financial resources and social networks impacted their ability to commission additional services to reduce the carer load.^59^ Patients with limited formal or informal support in the home were left with no choice but to seek hospital care:

Most people that call [911] don’t have any support at home. They live alone. Not being able to breathe and live alone, have no *one* to call, right? (HCP, p. 555)^59^

##### Difficulty accepting death

Across several studies, comments revealed a common thread of difficulty accepting death because it conflicted with an ingrained impulse to extend life. Participants described this tension in various ways. In the one pediatric study, an impulse to ‘do something’, (p. 226)^62^ even when comfort-focused care was clinically appropriate is described:

What I experienced at home was beautiful. . . but at the very last moment, I do not know what I felt, but I had to do something, and called for an ambulance (Carer, p. 226)^62^

In part, this action is associated with societal views of what constitutes a ‘(sic) good parent’,^62^ as the ‘(sic) saviour of their child.’(p. 225)^62^ Anotherstudy described how socio-cultural concepts influenced the decision to seek hospital care while enrolled in community based specialist palliative care:

One concept included the “hero,” which was invoked to describe patients who “don’t want to die” and associated aggressive measures, including hospitalization, with “reassurance” that they were taking every possible action to prolong life. (Primary study author quoting HCPs, pp. 554–555)^59^

Socio-cultural beliefs about the difficulty of accepting death were also described as shaping decisions to hospitalise residents in nursing homes. Specialist palliative care nurses noted that, although hospitalisation was often not beneficial for palliative patients,^54^ it continued to occur because of prevailing attitudes:

Some nursing homes. . .have a very good palliative approach. Others are still in that mindset of trying to keep people alive. (SCPC Nurse, p. 78)^54^

The concept also appeared in carers’ reluctance to use opioids due to concerns about hastening death, prompting hospital care for symptom management.^59,60,62^

##### Transitions as catalysts for misunderstanding

Healthcare professionals suggested that some patients and/or their carers lacked a clear understanding of the scope of services provided by palliative care when transitioning from hospital-based specialist teams to community-based specialist palliative care,^59,60,62^ and that some ‘(sic) come into hospice not having any idea what it’s about’ and are ‘(sic) expecting a miracle’. (HCPs, p. 554)^59^ Misunderstanding of palliative care is exemplified by this patient chart review quote:

Patients and/or caregivers were also found to have a misunderstanding of the hospice philosophy. . .including the goal to minimize further trips to the hospital. . .One caregiver brought a patient to the hospital for altered mental status because the caregiver thought hospice focused solely on wound care. . . (Author commentary on patient chart notes, p. 748)^56^

Healthcare professionals also suggested that patients and carers were often unaware that the curative treatment had ceased^60^ or that the patient was dying.^59,60^ It was suggested that when the patient and healthcare team goals of care are misaligned, patients are more likely to seek hospital based care:

I found that I have a lot of patients from a [medical center who] have not been told that they are dying [. . .] I think if there was better patient education on that end, it would eliminate a lot of [hospitalization] (HCP, p. 53)^60^

For patients with palliative care needs living in a nursing home, their transition to specialist palliative care may differ due to fragmented palliative care service delivery and miscommunication between nursing home staff around monitoring and adherence to end-of-life discussions and advance care planning.^54^ Even when patients requested not to be transferred to hospital, organisational policies typically led to ambulance calls during deterioration, challenging the provision of specialist care:

Unless you have got the documentation there, you actually can’t do anything else but send them back [to hospital]. (Nurse, p. 79)^54^

#### Individual determinants

The second set of third-order constructs focuses on individual determinants, illustrating how carers’ physical and emotional limits, patient and/or carer preferences and the patient’s clinical condition shape decisions to seek hospital care.

##### Threshold of carer capacity

Hospital use often occurred when patient deterioration coincided with carers reaching their physical, practical or emotional capacity to manage care at home. Witnessing deterioration of a patients’ physical or psychological condition was distressing for many carers, as exemplified by the language they used to describe deteriorating health, including; desperation for relief,^61^ ‘emergency’,^61^ ‘horrific’,^63^ ‘panic’,^59–61,63^ ‘crisis’^59,60^ and not being prepared for the patient to die.^63^ This emotive language explains why carers often instigated hospital use, even when it may be contradictory to the patient’s wishes to be cared for at home:

They understand [the wish of the patient is to die at home], but at a moment of crisis when they see a loved *one* [dying], it’s like all bets are off. . .(Spiritual Care Counsellor, p. 54)^60^

Unexpected deterioration prompting hospital use was most often described as unrelieved symptoms such as shortness of breath, pain and changes in cognitive status,^56,61,63–65^ or unexpected acute health events, such as falls and seizures:^61,63^

It’s a breathing problem. That’s why [caregivers] call 911 - they cannot get the patient comfortable breathing. (Nurse, p. 53)^60^

When I came [home] and saw [she was having a seizure], I said we have to call 911. . .it was horrific. . . (Carer, p. 262)^63^

Healthcare professionals suggested that any sudden unexpected deterioration of the patient between visits, such as accidents,^63^ exacerbation of other co-morbidities^59^ or necrotic pressure area^65^ often prompted them to recommend hospital transfer, as exemplified by this carer:

She fell out of bed and broke several vertebrae. She also needed stitches. I called the home hospice hotline first and when the nurse arrived, she advised me to call 911. (Carer, p. 263)^63^

In addition to examples of acute deterioration, caring was described as incremental and bore a physical and emotional burden as carers took on additional tasks, including transportation and physical assistance^56,65^ and then later medication administration.^59–61^ Managing medication was challenging for carers, even when they had received training from specialist staff, due to uncertainty around operating devices and fears that administering opioid medication might inadvertently hasten death:^59–61^

. . .so even though you teach [caregivers], when something happens and you tell them, well, you can give them morphine—“I can’t do it, I need somebody else to do it.” And [caregivers] just 9-1-1 it, because that’s what they’ve done their whole life. (HCP, p. 53)^60^

A carers threshold for keeping a patient at home may be exceeded when the demands of care surpass their skills and available support, forcing them to seek hospital care.^60,65^ For example, some quotes demonstrated a lack of confidence in managing symptoms, even where they had been given specific education or training to manage them.^60,61^

. . .even though the nurses had shown us how to use a nebuliser, we were overwhelmed with panic in that moment, so we made the decision to send him to the hospital (Carer, p. 4)^61^

This lack of confidence in managing unrelieved symptoms contributed directly to unplanned hospital presentations.

Carer capacity was also breached when the emotional distress of witnessing patient deterioration made prior guidance, such as contacting the palliative care service first or allowing the natural course of dying, no longer an acceptable option:

My sister told me she turned blue. [She] panicked. . .[she] called 911 right away. . . I think it’s harder when your loved *one* dies at home. You’re right there, you’re with the person everyday, just to watch them die it’s not a good feeling. (Carer, p. 262)^63^

The moment I saw her suffering, her whole body was shivering, I was afraid as I am not medically trained. The [homecare] nurse informed us to call them anytime in the event of emergency, but when you see your loved *one* is suffering, you are compelled to take immediate action. You have to arrange for hospital transfer so she could receive prompt medical attention. (Carer, p. 4)^61^

Further, carers and clinicians reported that a patient’s psychological needs often precipitated hospital use for acute health events, especially when they were highly distressed or anxious:^58,64,65^

Carer reports client is “anxious and at times this exacerbates her breathlessness” (Patient progress note, p. 10)^65^

These examples demonstrate the multiple pressure points carers face, highlighting how a range of emotional, practical and physical factors may prompt hospital use.

##### Hospital as a preferred place of care

There were several reasons why hospital was seen as the preferred place of care, even when patients were actively enrolled in a specialist community palliative care service. In many cases, it was perceived as the default choice,^55,60,61^ and is described as: ‘(sic) what we have always done’ (p. 445),^55^ or ‘(sic) where you go when you’re sick’ (p. 555).^59^ Although specialist community palliative care services often encouraged patients and carers to contact them first as part of their supportive care approach, repeated instances showed that patients and carers instead defaulted to acute care services:^55,57,61^

. . .information regarding how to contact the hospice nurse when issues arise and not to call 911 or go to the ED for symptom management is given to patients and caregivers. However, even with instruction to call the hospice nurse for symptom management issues, patients still access the ED (Study author, p. 447)^55^

There was a link between an expressed preference for hospital care and patients and carers having established relationships with the hospital-based health professionals over the course of illness.^57,60,63^ Some carers and patients felt safest in the presence of someone they trusted and had established a relationship with:

There may also be situations where there is still a strong relationship with the cancer center where [patients] were being treated, and wanting to go back there or feeling more of a connection with them than with the hospice team (HCP, p. 53)^60^

It was suggested that patients and carers were more likely to seek hospital care if they have not had time to establish rapport with the community team:

. . .If I get to know the patient in the last 24 h of life, he is unstable, in pain, breathing heavily - They don’t know me yet -sure, they will go to the hospital and he. . .will die there in the ER or in the intensive care unit (SCPC Nurse, p. 1673)^57^

Patient preferences for care in response to symptoms sometimes prompted direct requests for hospital care, even where there was no imminent threat to life, leaving the carer or health professionals with a sense that hospitalisation is, therefore, unavoidable:^55,61,63^

My father did not mind dying in the hospital, and he strongly preferred receiving medical care for his symptoms in the hospital. . .One day, he experienced abdominal bloating, prompting me to call the [community specialist palliative care] nurse. After assessing, she suggested waiting until the following day for her visit. But my father insisted, so I had to call the ambulance.” (Carer, p. 3)^61^

### Expressing the synthesis

**Figure 3.**
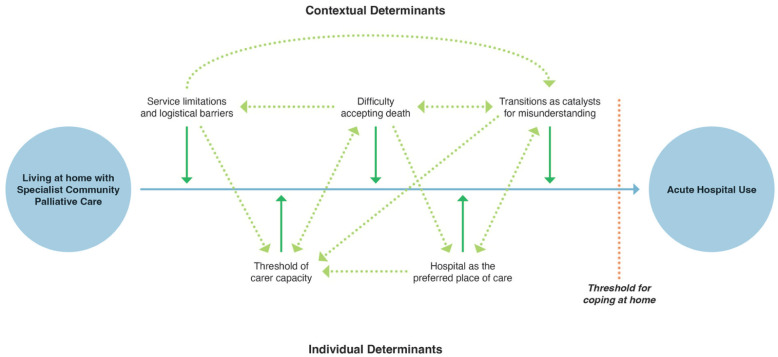
Conceptual map of reasons for hospital use in specialist community palliative care.

Hospital use emerges as a coping strategy when individual vulnerabilities (clinical decline, carer capacity, personal care preferences) intersect with contextual constraints (service gaps, logistical barriers, mismatched expectations, difficulty accepting death), creating a reinforcing cycle of perceived safety and systemic dependency. Figure 3 depicts that while each third-order construct functioned independently as a direct reason for hospital use, their interconnections added layers and pathways that ultimately culminate in crossing the threshold of coping at home, leading to hospital use. Carers and healthcare professionals often focused on the critical moment when reporting why the decision to hospitalise was made in the included studies. Through translation and synthesis, a broader understanding emerges, where decisions to hospitalise are shaped by the cumulative effect of individual and contextual pressures, with hospital use often functioning as a coping strategy when these pressures converge. These pressures, while not always explicitly acknowledged, play a significant role and warrant careful consideration.

## Discussion

The meta-ethnography found that hospital use for individuals receiving palliative care in the community is rarely driven by clinical need alone. Instead, it reflects a convergence of emotional, temporal, relational, and systemic pressures. The capacity of patients and carers to cope is dynamic and evolves, influenced by both contextual and individual determinants. The decision to seek hospital care for this population, therefore, emerges from this complex interplay of factors.

The studies in this meta-ethnography often presented themes in isolation, treating physical symptoms, carer distress and other reported factors as separate reasons for hospital use. For example, acute clinical symptoms marked an automatic tipping point for specialist community palliative care patients to seek hospital care.^55,58–61,63^ Additionally, recent quantitative studies often document the reason for hospitalisation as the presenting symptom or medical event at admission, isolating these as targets for improving symptom control in the community.^33,38,66,67^ However, reciprocal translation revealed a richer context. In some accounts, symptoms appeared to be secondary to caregiver distress;^60,61^ in others, symptoms themselves drove admission;^58,59,63^ and in yet others, symptoms exposed gaps such as missing equipment or service limitations.^60,61,65^ This model challenges the assumption that hospital use is a straightforward reaction to acute symptoms or a carer struggling to cope, suggesting instead that it is often a layered response to cumulative strain.

One example of this interplay is carers seeking hospital care after struggling with clinical tasks such as using medical equipment^61^ or recognising when to administer opioid analgesia.^55,60^ The default assumption might be that this reflects a knowledge gap best addressed through education. However, this example can be understood in the context of individual and contextual determinants, including emotional burden (e.g. fear of doing harm or witnessing severe symptoms),^59–61,63–65^ inadequate support as the patient transitions from active treatment to palliative care,^54–56,59,60,62^ and palliative care service limitations (e.g. service scope, provider scope of practice, operating hours, or emergency response time).^54,56,60,61,63,65^ Increasingly, many carers are older adults who are frequently managing their own health, isolation, and mobility challenges.^68–70^ In such cases, an additional education session is unlikely to address their individual needs in full, with effective solutions requiring a more holistic and multidisciplinary approach. Models such as Chapman et al.’s symptom management framework highlight the need to address both psychological distress and practical symptom control in palliative care, integrating these through shared decision-making.^71^ Holistic models of care should be standard practice in specialist community palliative care to address hospital use.

This complexity is also evident when a patient or carers preference for hospital-based care is cited as a direct reason for hospital use.^57,60,63^ Carers in the included articles perceived hospitals to be places of safety and recovery,^57,59–61,63^ confirming the findings from other studies.^37,72,73^ However, by demonstrating linkages between emotional overwhelm,^56,64^ carer burden,^60,63,65^ difficulty accepting death,^54,59,62^ and limited or fragmented palliative care service support,^61,65,70^ this meta-ethnography enhances the understanding of why carers perceive hospitals as places of safety during uncertainty.

There is evidence that the full benefits of community palliative care are only realised after 3–4 months of consistent access and service, however, not all patients are afforded this opportunity.^74,75^ Accordingly, this meta-ethnography found that patients who transferred to specialist community palliative care in the last days or hours of life may lack the opportunity to build rapport and trust with community clinicians,^57^ and were more likely to seek hospital care in a crisis.^57,60,63^ Restrictive specialist palliative care referral policies, such as the US requirement for a prognosis of fewer than 6 months with cessation of all active treatment to qualify, make it harder for patients to be referred earlier.^74,76^ Additionally, referral timing varies by disease type, with specialist palliative care better integrated into cancer services than other progressive life-limiting illnesses.^59,74^ Earlier referral and structured transitions may help mitigate these two key contextual determinants, which in turn, could directly reduce the incidence of unplanned hospital use.

Another example of the interplay of individual and contextual determinants is evident where carers report bypassing specialist palliative care service emergency consultations, opting instead to go directly to the hospital or calling an ambulance.^55,61,63^ This meta-ethnography found multiple accounts of a lack of timely access to medical equipment, mobility aids and formal carer support such as respite care.^61,65,70^ Limited access to these resources and increasing care needs can quickly erode a carer’s resilience.^77,78^ Carers’ descriptions in the included studies of experiencing ‘*panic*’^59–61,63^ and *distress*^55,56,64,65^ were linked to situations where the patient experienced unexpected and/or acute symptoms or events, and the carer felt compelled to act. Decisions made in this heightened emotional state were more likely to result in hospital use. As caring responsibilities predominantly fall on informal caregivers,^79^ formal patient and carer social supports may be most effective in addressing carers unmet needs.^61,79^

Healthcare professionals also described situations where patients and carers transitioning home from hospital-based care were unaware of the terminal nature of illness or misunderstood the goals and scope of specialist palliative care.^54,56,59,60,62^ This knowledge gap may contribute to patients and carers not contacting the palliative care service in an emergency: A 2025 study of a community-based rapid response specialist palliative care intervention was able to increase the time patients spent at the end-of-life in their own home by providing timely, practical and emotional support to carers when they were most frightened or scared.^80^ While this type of intervention can prevent hospital use, the impact is limited if patients and carers are unaware of how the service can support them.

While hospital care is often entirely appropriate^58,72,81^ this meta-ethnography explores the complexity behind unplanned hospital use, rather than suggesting it should be avoided. Many of the quotes reflected this: In some cases, healthcare professionals recommended hospital transfer based on the patient’s condition and the scope of the palliative care service.^54,59,63^ One study described a carer seeking hospitalisation for a patient after the specialist community service did not provide a clear explanation for withholding analgesia.^61^ Further, some patients may prefer to receive care at home until a certain point, then transition to the hospital for end-of-life care or as their condition changes,^82^ a sentiment reflected in several participant quotes.^61^

Nonetheless, the examples discussed in this meta-ethnography illustrate how carers report, and patients are perceived as, turning to hospital care as a way of coping with the cumulative pressures of physical condition, emotional distress, systemic constraints, and societal expectations, not merely as a response to one catalyst.

### Strengths and limitations

Employing a systematic and rigorous meta-ethnographic approach is a strength of this review. Incorporating primary quotes from study participants along with the original study author interpretations enabled the meta-ethnography process to generate new insights and interconnect findings.^42^ While the voices of carers and healthcare professionals are well represented, the absence of patient perspectives remains a limitation of this study and the broader evidence base. This likely reflects the challenges of engaging vulnerable populations in qualitative research.^83,84^ Future studies could adopt flexible, ethically considerate approaches, such as narrative or participatory methods, and consider incorporating quantitative tools like surveys to more feasibly and robustly capture patient experiences. The included studies represent a diverse range of developed countries, with differing healthcare systems and models of specialist community palliative care services, which impact the supports provided and experience of patients and their carers.

Because reasons for hospital use were often a secondary or incidental finding, some sources contributed a minimal amount of qualitative data, which is a limitation of the meta-ethnography approach adopted to synthesise the available data. Two of the studies^56,64^ did not report any primary quotes relating to reasons for acute care use, compromising the depth of synthesised data, as only the primary study authors’ interpretation is presented. While three of the studies included nursing homes in their inclusion population, only one of these studies^54^ presented data specifically originating from a nursing home environment and therefore it was not possible in this meta-ethnography to generate insights on the delivery of specialist community palliative care in this setting. Finally, several participant quotes were relevant to more than one theme, reflecting the complex and interrelated nature of experiences in specialist community palliative care. In such cases, quotes were categorised relative to the most dominant or contextually relevant theme. Despite these limitations, there was a high concordance between the carers and healthcare professionals represented in the available data.

## Conclusions

Hospital use in specialist community palliative care is rarely a simple clinical decision; rather, it reflects a complex coping response shaped by cumulative individual pressures, emotional burden and systemic limitations. This meta-ethnography highlights how individual and contextual factors interact to influence decision-making. While there is no evidence-based model for specialist community palliative care, these findings offer practical considerations for services, highlighting the need for holistic, multidisciplinary approaches that address both contextual and individual determinants of hospital use. While hospitalisations in palliative care patients can be unavoidable and appropriate, there may be opportunities for specialist services in the hospital and community to be more responsive to the collective needs of the service end-users. Particularly at critical time points such as palliative care referral and transitions from hospital to community. Further, there is a need for recognising and supporting carers’ emotional, physical, and relational needs through practical supports, accessible counselling and respite, and timely, effective management of patient symptoms and needs. By responding to these layered determinants, services may reduce acute care utilisation and enable patients and carers to spend more time in their preferred place of care. Further research is needed to determine the most effective models of care. These models of care need to be patient-centred and support their preferences and needs, as well as supporting and empowering their carer(s) to confidently manage them at home. Addressing the absence of patient perspectives through ethically considerate and accessible methodologies will be essential to strengthening future evidence and ensuring a more complete understanding of hospital use at the end of life.

## Supplemental Material

sj-docx-1-pmj-10.1177_02692163261418625 – Supplemental material for Reasons for patients in high income countries accessing hospital care while receiving specialist community palliative care: A systematic review and meta-ethnography Supplemental material, sj-docx-1-pmj-10.1177_02692163261418625 for Reasons for patients in high income countries accessing hospital care while receiving specialist community palliative care: A systematic review and meta-ethnography by Norah Elvidge, Melanie Rolfe, Karen Smith, Hannah E. Carter and Jane L. Phillips in Palliative Medicine

sj-docx-2-pmj-10.1177_02692163261418625 – Supplemental material for Reasons for patients in high income countries accessing hospital care while receiving specialist community palliative care: A systematic review and meta-ethnography Supplemental material, sj-docx-2-pmj-10.1177_02692163261418625 for Reasons for patients in high income countries accessing hospital care while receiving specialist community palliative care: A systematic review and meta-ethnography by Norah Elvidge, Melanie Rolfe, Karen Smith, Hannah E. Carter and Jane L. Phillips in Palliative Medicine
