# Supplementary material for: Reasons for patients in high income countries accessing hospital care while receiving specialist community palliative care: A systematic review and meta-ethnography
Source: Palliat Med. 2026 Feb 25;40(6):705–21. doi: 10.1177/02692163261418625 (PMC13221571; doi:10.1177/02692163261418625)
Supplement: sj-docx-1-pmj-10.1177_02692163261418625 – Supplemental material for Reasons for patients in high income countries accessing hospital care while receiving specialist community palliative care: A systematic review and meta-ethnography [file sj-docx-1-pmj-10.1177_02692163261418625.docx]

Supplemental File 1.

Search terms for the Systematic Review and Meta-Ethnography

| "community care", "care at home", "home health nursing", "home care", "home based care", "home nursing", "home care services", "home health care", "community nursing" |
| --- |
| "palliative care", "end of life care", “terminal care", "supportive care", "palliative therapy", "hospice care", "life limiting disease", "life limiting illness", "life limiting condition", "terminal illness", "terminal disease", "advanced disease", "advanced malignancy" |
| "Hospital admission", "hospital readmission", "unplanned hospital admission", "unplanned hospital readmission", hospitali?ation, "hospital stay", "acute admission", "emergency department", ED, "accident and emergency", A&E, "inpatient ward", "hospital use", "hospital presentation" |
| 1 and 2 and 3 |
| Limiters - Publication Date: 20140101-20241231 and English language |
| *Note: slight variations in search terms were used to cater to different databases.* |
