# Supplementary material for: Reasons for patients in high income countries accessing hospital care while receiving specialist community palliative care: A systematic review and meta-ethnography
Source: Palliat Med. 2026 Feb 25;40(6):705–21. doi: 10.1177/02692163261418625 (PMC13221571; doi:10.1177/02692163261418625)
Supplement: sj-docx-2-pmj-10.1177_02692163261418625 – Supplemental material for Reasons for patients in high income countries accessing hospital care while receiving specialist community palliative care: A systematic review and meta-ethnography [file sj-docx-2-pmj-10.1177_02692163261418625.docx]

Supplemental File 2: JBI Quality Assessment

| **Question Number** | **Q1** | **Q2** | **Q3** | **Q4** | **Q5** | **Q6** | **Q7** | **Q8** | **Q9** | **Q10** |
| --- | --- | --- | --- | --- | --- | --- | --- | --- | --- | --- |
| **Cross Sectional Study Design (With qualitative elements)** | | | | | | | | | | |
| DeAngelis DeAngelis and Lowry (2021) | Yes | Yes | N/A | Yes | Yes | No | No | N/A |  |  |
| Batchelor (2015) | Yes | No | Yes | Yes | No | No | Yes | Yes |  |  |
| **Qualitative Study Design** | | | | | | | | | | |
| Yoon, Goh (2024) | No | Yes | Yes | Yes | Yes | Unclear | Unclear | Yes | Yes | Yes |
| Halevi Hochwald, Radomyslsky (2022) | No | Yes | Yes | Yes | Yes | No | No | Yes | Yes | Yes |
| Papadatou, Kalliani (2021) | Yes | Yes | Yes | Yes | Yes | No | No | Yes | Yes | Yes |
| Ward, Sixsmith (2021) | No | Yes | Yes | Yes | Yes | Yes | Yes | Yes | Yes | Yes |
| Russell, Baik (2019) | No | Yes | Yes | Yes | Yes | No | No | Yes | Yes | Yes |
| Phongtankuel, Scherban (2016) | No | Yes | Yes | Yes | Yes | No | No | Yes | Yes | Yes |
| Phongtankuel, Paustian (2017) | No | Yes | Yes | Yes | Yes | No | No | Yes | Yes | Yes |
| Champion (2015) | No | Yes | Yes | Yes | Yes | No | No | Yes | Yes | Yes |
| Lane and Philip (2015) | No | Yes | Yes | Yes | Yes | No | No | Yes | Yes | Yes |
| Hatcher, Harms (2014) | No | Yes | Yes | Yes | Yes | No | No | Yes | Yes | Yes |
